# Supplementary material for: Are carbon emissions trading and green financial instruments synergistic? -Comprehensive quantitative research based on content analysis
Source: PLoS One. 2024 Mar 7;19(3):e0298601. doi: 10.1371/journal.pone.0298601 (PMC10919601; doi:10.1371/journal.pone.0298601)
Supplement: S1 Data — (ZIP) [file pone.0298601.s001.zip › Data/地级市Z_F工作报告.zip!/░▓╗╒/═¡┴Ω╩╨/└┤╫╘╣1⁄2╓┌║┼í╛┬φ┐╦╩2╛▌═°í┐_2016.docx]

各位代表：

　　现在，我代表市人民政府，向大会报告工作，请予审议，并请市政协委员和其他列席人员提出意见和建议。

　　一、2015年和“十二五”工作回顾

　　过去的一年，是铜陵历史上极不平凡、具有特殊意义的一年。全市人民在省委、省政府和中共铜陵市委的坚强领导下，全面贯彻党的十八大和十八届三中、四中、五中全会精神，深入贯彻习近平总书记系列重要讲话精神，牢牢把握稳中求进工作总基调，主动适应经济发展新常态，顶住经济持续下行压力，统筹推进各项工作，较好地完成了市十五届人大四次会议确定的各项目标任务，全市经济稳中有进、稳中向好，社会大局和谐稳定。

　　预计，全年实现地区生产总值721亿元（不含枞阳县，下同），增长10.1%，增幅居全省第四位。财政收入135亿元，增长2.1%，其中税收收入占比84.7%，高于全省平均水平11.4个百分点。全社会固定资产投资871亿元，增长13.5%，高于全省平均水平0.8个百分点。社会消费品零售总额208.8亿元，增长10.5%。全市常住居民人均可支配收入28740元，增长9.8%。新增就业2.2万人。居民消费价格上涨1.2%。人口自然增长率2.81‰。节能减排实现年度控制目标。

　　一年来，我们主要做了以下工作：

　　第一，千方百计稳增长。认真落实中央和省系列稳增长政策，及时研究出台稳增长调结构促转型具体措施，着力稳定实体经济。强化问题导向，深化“暖企行动”，扎实开展“三百”活动。创新支持企业发展方式，组建产业投资、创业投资和风险投资三支基金。累计减免企业税收9.5亿元。拨付扶持发展资金超过5亿元。设立过桥续贷资金8.6亿元，协调金融机构动态认领企业252户，新增贷款87亿元。深化政银担合作，累计金额27.7亿元，位列全省第二。积极利用资本市场，完成直接融资35.9亿元，新增“新三板”挂牌企业6家。争取国家专项建设基金10.2亿元，省级代发债券额度44.1亿元，有效化解了债务风险，促进了经济建设。

　　着力扩大有效投入，开展“重大项目谋划建设年”活动。全年新开工亿元以上项目86个、竣工92个，开工率、竣工率均创历史最好水平。京福高铁、宁安高铁，铜南宣高速、铜陵长江公铁大桥及连接线建成通车；省道320、321，朱永路，滨江大道全线贯通。有色“奥炉”改造、皖能第二台百万千瓦机组、泰新年产10万辆新能源汽车等一批重大产业项目顺利推进。积极对接国家重大工程包，38个重大项目列入省计划笼子，G3高速铜陵长江替代桥前期工作取得重大进展。加强招商引资工作，引进沃特玛电池组、万达广场等10亿元以上项目4个、5亿元以上项目20个，在建省外投资亿元以上项目实际到位资金412.9亿元，实际利用外商直接投资2.23亿美元。累计拆除违章建设12万平方米，征收房屋145万平方米、土地8492亩，为项目建设提供了有力保障。

　　第二，加快推动“调转促”。深入实施创新驱动战略。新认定国家高新技术企业31家，新增省级工程技术中心4家、院士工作站1个，新引进高层次科技人才团队3个。全社会研发经费占GDP比重2.9%，居全省第2位。万人发明专利拥有量7.86件，居全省第4位。推进大众创业、万众创新，打造众创空间15家。强化转型平台建设，资源型城市考核蝉联全国榜首，国家节能减排示范市绩效考核位列全国优秀等次第三名。研究出台了“千百十亿”工程实施意见，推动产业向中高端发展。大力改造传统产业，完成技改投资333亿元，增长39.6%。加快发展战略性新兴产业，新兴产业产值占规上工业比重由30%提高到32.9%，占比居全省首位；铜基新材料产业成为全省首批战略性新兴产业集聚基地，狮子山开发区申报国家高新区取得重大进展。加快发展服务业，增加值占GDP比重达到29.8%，提高2.8个百分点，新增省级服务业集聚区2个，文化产业投资公司组建运行。加快发展港口经济，港口吞吐量突破8000万吨，集装箱突破4万标箱；“圈区管理”废七类集装箱进口转关申报取得进展，铜陵口岸获批建设进境粮食、水果指定口岸，铜陵港跻身国家主要港口行列。

　　第三，推进城乡大建设。精心组织实施城乡建设计划，全年完成投资60亿元。市政设施建设大踏步推进，新建改造城市主次干道15条，北京西路下穿等节点工程提前竣工，铜官大道等沿线区域环境大幅改善，城区断头路、丁字路基本打通；新建2座大型货车停车场，新增城区停车位1100个，新开通公交线路4条，新投放公交车58台；改建供水管网42公里，燃气管道48.7公里；改造了长江新村、五松新村等33个老旧小区，改善了6万居民的居住环境质量；推进智慧城市建设，建成全市统一的云计算数据中心，成为“宽带中国”示范城市。社会事业项目建设大获丰收，新建改造了市文化馆、五松山剧场、板栗山儿童公园、老年学习活动中心等一批项目，建成了一批全民阅读示范点，全国首个书馆一体的图书馆新馆、新华书店对市民开放。东部城区建设有力推进，大通古镇改造已具雏形。美丽乡村建设不断扩面，16个省级中心村、20个市级中心村面貌一新，凤凰山村成为首批中国乡村旅游模范村。大力提升城乡环境质量，实施大气污染整治项目1300多个，PM10平均浓度下降16.6%，南部城区扬尘治理形成长效机制；中心城区“三大水系”治理取得积极成效，正在成为城市的生态休闲廊道；持续开展“三线三边三小”环境整治，拆除小码头、非码头设施64处，20公里的滨江岸线整治任务提前完成；新增造林2.87万亩，绿化提升392万平方米，恢复矿山植被3000亩，建成森林长廊和城市绿道40公里，创建国家森林城市取得明显成效。

　　第四，深化改革增活力。完成年度改革任务71项，基本实现预期目标。稳步推进行政体制改革，全面完成政府机构改革。在全省率先建立运行市县乡三级政府权责清单，243项行政审批事项实现网上办理。强力推进“多规合一”试点，按照多规统一融合的思路，改革建设项目行政审批流程，数字化平台基本建成。“三证合一”、“一照一码”等商事制度改革全面推行。公务用车改革顺利实施。农村土地承包经营权确权颁证基本完成，经营权抵押贷款取得突破，城乡一体化综合配套改革深入推进。城市排水一体化PPP试点项目挂牌。国有资本收益上缴比例提高到15%。政府购买服务规模突破2亿元，增长44%。降低非居民供电供气价格，减轻企业负担2.2亿元。扎实推进社会事业改革，教联体、医联体建设不断深化，义务教育阶段实现“阳光招生”、“阳光分班”，公立医院改革走在全省前列，社区“三减一加强”成果得到巩固。

　　第五，改善民生促和谐。认真落实各项惠民政策，财政民生类支出达92亿元，占总支出的83.8%。精心组织实施38项民生工程，绩效评价连续三年位列全省先进。棚户区改造力度前所未有，友好、天桥、源北等片区4570户居民告别棚户区，圆了“安居梦”。加大安置房、保障房建设力度，1.3万户回迁居民搬入新居或分到新房。完成农村危房改造3200户。实施购置住房补贴等政策，房屋销售面积增长6.4%，增幅位居全省前列。不断完善社会保障体系，城乡养老待遇全面提标，大病医疗保险实现全覆盖，城乡低保、特困人员供养标准提高10%以上，“救急难”工作成为全国试点。完成义务教育学校标准化建设，开工兴建市特殊教育学校，成立了职业教育联盟。成功申报创建国家公共文化服务体系示范区。市立医院创建“三甲”综合医院通过省级评审，人民医院外科楼、中西医结合医院主体完工。全民健身运动广泛开展，市体育中心投入市场化运营。新获得一批“国字号”荣誉，成为全国文明城市，未成年人思想道德建设工作先进城市，无障碍建设示范市，幸福家庭示范市。国家卫生城市复审获得全国优秀，全国双拥模范城市完成考核。安全生产形势稳定向好。依法信访有效落实。城市社区居委会换届选举圆满完成。“平安铜陵”建设扎实推进，社会治安综合治理成为全省先进市。社会科学、新闻出版、广播影视、文学艺术、统计、对台、外宣、档案、地方志、供销、气象、地震、消防、兵役等工作得到加强，民族宗教、外事侨务、妇女儿童、红十字等事业取得新成绩。

　　过去的一年，我们切实加强法治政府建设。聚焦“忠诚、干净、担当”，扎实开展“三严三实”专题教育，推进巡视、审计和督查发现问题的整改。坚持依法行政，建立了市政府常务会议学法制度、重要文件解读制度。规范政府系统重大事项决策行为，严格合法性审查。加大政府信息公开力度，网上信息公开工作走在全国前列。自觉接受人大法律监督、工作监督和政协民主监督，按时办结人大代表建议191件、政协提案302件。大力开展“政府管理提升年”活动，建立健全70余项管理制度，基层基础管理得到规范，建设投资等重点领域管理明显加强，管理体制机制进一步完善。严格执行中央八项规定，严肃财经纪律，“三公”经费下降16.9%。深入开展查处发生在群众身边的“四风”和腐败问题专项行动，严肃查办了一批违法违纪案件。

　　过去的一年，枞阳县经济社会发展取得长足进步，生产总值达到195亿元，重点建设进展顺利，人民生活继续改善。区划调整实施以来，县委、县政府坚决贯彻中央批复精神，认真落实省委、省政府决策部署，同心同德、步调一致，广大人民群众坚决拥护，广大干部职工积极支持，各项工作有力有序推进。

　　各位代表！2015年各项目标任务的完成，为“十二五”画上圆满句号。回首过去的五年，以行政区划重大调整为标志，我们跨入市域新时代；以京福高铁、宁安高铁开通为标志，我们跨入高铁新时代；以荣膺第四届全国文明城市等为标志，我们跨入文明建设新时代；以资源型城市转型、节能减排、循环经济等试点示范工作走在全省全国前列为标志，我们跨入转型发展新时代；以铜加工材产量超过电解铜产量为标志，我们跨入铜业延伸加工新时代。区域发展迎来新格局，转型升级迈上新平台。

　　五年来，全市综合实力跃上新台阶。GDP总量是“十一五”的2.04倍（含枞阳县，下同）。财政收入总量是“十一五”的2.03倍。累计完成固定资产投资4013亿元，是“十一五”的2.9倍。

　　五年来，转型发展实现新突破。战略性新兴产业产值翻两番，规模达650亿元，占规上工业比重由12.9%提高到28.8%。市场主体更富活力，民营经济占比由54.8%提高到65.6%。枞阳县连续三年成为全国粮食生产先进县，成功创建全国国土资源节约集约模范县。

　　五年来，改革开放迈出新步伐。完成改革任务186项，理顺了投融资、招投标和土地管理等领域的体制机制，社区“三减一加强”、棚户区改造“房票”安置、警务机制、医联体和教联体等改革举措富有特色，卓有成效。积极融入长江经济带建设，对外开放广度深度进一步拓展。

　　五年来，事业发展呈现新气象。城市功能不断完善，建成区面积由69.2平方公里增加到81.2平方公里，主城区功能品质大幅提升。美丽乡村成为我市新的魅力板块。各项事业持续进步。民主法制建设不断加强。平安城市建设摘取国家最高荣誉“长安杯”。

　　五年来，人民生活达到新水平。城镇居民和农村居民人均可支配收入分别达到30050元、10810元。强力推进环境治理修复，全市人居环境显著改善，人民群众的自豪感、幸福感和归属感不断提升。

　　各位代表！五年的成绩来之不易，这是省委、省政府和市委科学决策、正确领导的结果，是历届班子坚持不懈、奋发努力的结果，是全市人民团结拼搏、砥砺奋进的结果。在此，我代表市人民政府，向全市广大工人、农民、知识分子、干部、离退休老同志，向驻铜解放军指战员、武警官兵和政法干警，向各民主党派、各人民团体和社会各界人士，向关心、支持铜陵发展的省直各部门和海内外友好人士，表示衷心的感谢，并致以崇高的敬意！

　　各位代表！五年的开拓奋进，五年的辉煌成就，让我们深切地体会到，推进铜陵发展，必须坚持把转型发展作为主攻方向。牢记发展是硬道理、转型是硬任务，保持转型发展的战略定力，充分发挥比较优势，既毫不动摇地狠抓发展第一要务，又坚定不移地推进绿色发展、低碳发展和循环发展。必须坚持把改革创新作为强大动力。始终围绕主题主线、民计民生、和谐稳定来谋划改革，推动思想解放，理顺体制机制，扩大对外开放，承接产业转移，推进创新创业，不断积蓄持续发展的新动能。必须坚持把宜居宜业作为不懈追求。突出环山、滨江、湿地特色，坚持“山水林田湖”共生共存，促进城乡协调发展，坚定不移地打造宜居宜业的山水园林城市。必须坚持把共建共享作为发展原则。坚定人民至上的价值取向，始终把保障和改善民生作为一切工作的出发点和落脚点，着力抓好民生工程、完善公共服务、促进公平正义，极大焕发广大人民群众建设美好家园的热情。必须坚持把作风建设作为根本保障。以好的作风谋发展、抓落实、树导向，增强干部执行力，以作风建设的新常态引领经济发展新常态。

　　与此同时，我们更清醒地认识到，在转型发展进程中，还面临诸多挑战：产业转型升级和供给侧结构性改革任重道远，新旧发展动能的转换还有一个较长过程；资源环境容量约束加剧，节能减排、环境治理和生态建设压力空前；区划调整后的城乡发展不平衡、不协调矛盾突出，农村发展和扶贫脱贫任务十分繁重。当前，经济下行压力持续加大，有色、冶金、建材等传统产业进入深度调整期，主导产品价格连续49个月低位徘徊。实体经济困难增多，少数企业资金链断裂，部分企业处于停产或半停产状态。建设领域特别是房地产市场调整新旧矛盾交织，深层次问题水落石出，化解难度不断加大。新开工重大项目减少，投资增长后劲不足。财政增收艰难，刚性支出不断增加。地方债务风险化解任务依然很重。潜在金融风险不容忽视。极少数干部忧患意识、担当意识不强，为官不为、执行力不强的现象还不同程度存在。我们必须高度重视，采取切实措施，认真加以解决。

　　二、“十三五”时期的主要目标和任务

　　“十三五”时期，是全面建成小康社会的决胜阶段，也是铜陵加快转型发展的关键时期。未来五年，国际国内经济深度调整，新一轮科技革命和产业革命蓄势待发，创新引领发展成为时代潮流，新的增长动力正在孕育形成，我国经济长期向好的基本面没有改变。长江经济带建设进入全面推进阶段，有利于我市改善发展支撑条件，加快产业升级，增创综合发展优势；区域发展格局的重大变化，极大拓展了城市建设与发展空间，有利于推动南北互动，提高资源配置效率；区域交通枢纽地位日益凸显，有利于增强我市要素集聚能力，全方位扩大对外开放。特别是历经60年的持续奋斗，我市综合经济实力大为增强，城市综合竞争力大有提高，发展环境日益优化，发展路径更加清晰，加快铜陵发展大有可为。我们必须坚定发展信心，抢抓发展机遇，勇于担当，主动作为，适应新常态、把握新常态、引领新常态，奋力把铜陵发展推向新的更高水平。

　　我市“十三五”时期发展的指导思想是：高举中国特色社会主义伟大旗帜，全面贯彻党的十八大和十八届三中、四中、五中全会精神，深入贯彻习近平总书记系列重要讲话精神，紧紧围绕“四个全面”的战略布局，全面践行创新、协调、绿色、开放、共享的发展理念，按照“抢占竞争制高点、争当发展排头兵”的目标定位，以提高经济发展质量和效益为中心，坚持走创新转型、绿色转型、文明转型之路，集中力量打好转型升级、环境治理、深化改革、扶贫脱贫攻坚战，加快供给侧结构性改革，形成引领经济发展新常态的体制机制和发展方式，统筹推进经济建设、政治建设、文化建设、社会建设、生态文明建设和党的建设，加快建设富有实力的新型工业化城市、富有活力的创新创业城市、富有魅力的山水园林城市和富有向心力的人民满意城市。

　　今后五年经济社会发展的主要目标任务是：全面建成小康社会，实现更高质量、更有效率、更加公平、更可持续的发展。

　　———转型发展走在前列。经济保持中高速增长，产业迈向中高端水平，到2018年提前实现地区生产总值比2010年翻一番，到2017年提前实现城乡居民人均收入比2010年翻一番；到2020年工业增加值突破1000亿元，战略性新兴产业产值占规上工业总产值比重达30%以上，服务业增加值占地区生产总值比重力争达到35%，现代农业特色彰显，新产业新业态引领作用凸显，综合实力显著增强。创新驱动成为发展主引擎，研发经费支出占地区生产总值比重达3%，争创国家级创新型城市。继续在循环经济、节能减排等领域积极探索创新，建成全国资源型城市转型示范市。

　　———城乡区域协调发展。国土空间主体功能定位明确，县区职能任务更加明晰，形成布局科学、分工合理、各具特色、互动共进的发展格局。新型城镇化和城乡一体化步伐加快，常住人口城镇化率达60%以上，城乡和区域之间居民收入水平、基础设施水平、基本公共服务均等化水平等方面差距进一步缩小。“多规合一”全面推行，城市规划建设管理水平显著提升。

　　———生态环境持续改善。“山水林田湖”共生共存、共促共进的理念牢固树立，系统完整的生态文明制度体系基本建立。环境综合整治力度持续加大，全社会环境意识持续增强，生产方式和生活方式的绿色、低碳水平持续提升。成功创建国家森林城市，森林覆盖率达27%，生态城市发展健康指数全国领先，长江铜陵段生态环境明显改善。能源资源使用效率不断提高，单位GDP能耗持续下降。主要污染物排放得到有效控制，大气、水体、土壤污染得到有效治理，城市空气质量优良天数比率进一步提高，城乡环境质量明显改善。

　　———人民生活更加幸福。坚持把文明创建作为城市转型的重要内容和精神内核，中国梦和社会主义核心价值观更加深入人心，公民文明素质和社会文明程度显著提高。现行标准贫困人口全部脱贫，中等收入人口比重提升。就业岗位更加充分，创新创业蔚然成风，劳动关系更加和谐。教育、文化、卫生、社保、养老、住房等基本公共服务体系更加健全，人民生活水平普遍提高，继续保持全省先进行列。

　　———治理体系趋于完善。着力构建共建共享的社会治理格局，重要领域和关键环节改革取得决定性成果，治理体系和治理能力现代化取得重要进展。人民民主更加健全，平安建设基础更加牢固，法治建设体系更加完善，最大限度增加和谐因素，增强社会发展活力，确保全市人民安居乐业、社会安定有序。

　　实现上述目标任务，要突出“转型发展、协调发展、拥江发展”三大战略。要把握五大重点，即坚持以铜为基，加快产业转型升级；坚持以港兴市，推进港城一体发展；坚持以绿为核，加强生态文明建设；坚持以城带乡，提升基本公共服务；坚持以文化人，汇聚强大精神动力。具体任务是：

　　（一）建设富有实力的新型工业化城市。加快新型工业化进程。全面落实《中国制造2025》和全市“调转促”行动计划，发展壮大战略性新兴产业，提升传统产业生命力和竞争力，深入实施新一轮技术改造，推进“千百十亿”工程建设，完成技改投资1000亿元以上，力争建成2个千亿元产业、7个百亿元产业和一批10亿元产业，力争铜陵有色跻身世界500强，铜化集团进入全省20强行列。加快发展现代农业，构建现代农业产业体系，培育新型农业经营主体，拓展现代农业功能，着力打造区域最具特色的现代农业示范基地。大力发展生产性服务业，优化提升生活性服务业，建设省级现代服务业集聚区15个，构建功能完善、布局合理、服务高效、供给良好的现代服务业体系。强化园区载体、政策平台、招商体系、资本市场和质量品牌建设，奋力将经济技术开发区建成全国一流的开发区，继续创建国家级试点示范，为产业转型提供强大支撑。

　　（二）建设富有活力的创新创业城市。坚持创新驱动，深化科技体制改革，强化企业创新主体地位和作用，加强政府服务创新能力建设，推进大众创业、万众创新，构建发展新优势。新认定高新技术企业70家，新增城镇就业12万人，全市高技能人才总量达4万人。全面深化改革，深入开展行政体制改革、国企国资改革，优化经济发展环境，积极推进改革试点试验，培育发展新动力。全方位扩大开放开发，着力融入长三角经济圈和合肥都市圈，畅通大开放通道，完善大开放平台，全面提升开放型经济水平，构建发展新格局。大力发展文化旅游产业，不断丰富文化旅游业态，增添发展新动力。

　　（三）建设富有魅力的山水园林城市。尊重城市发展规律，加快建设皖中南中心城市。修编完善城乡规划体系。推进区域和城乡协调发展，科学开发江北区域，优化提升江南区域，推进城乡一体化发展，建设美丽乡村。全面推进绿色转型。筑牢生态安全屏障，加大环境治理力度，推动形成绿色发展方式和生活方式，积极争创国家生态文明示范区。

　　（四）建设富有向心力的人民满意城市。增进人民福祉，促进人的全面发展。提前打赢扶贫脱贫攻坚战，实现农村贫困人口全部脱贫，确保全面如期建成小康社会。全面提高社会事业发展水平，加大对新城区、薄弱领域的投入力度。深入推进教育现代化建设，完善公共文化服务体系，推进健康铜陵建设，贯彻人口均衡发展战略，基本社会保障覆盖率超98%。完善住房保障体系，全面完成棚户区改造任务。打造文明和谐社会。完善社会治理，全面推进依法治市，深化“平安铜陵”建设，持续提升城乡文明程度，不断提高人民群众的获得感、认同感和幸福感。

　　三、2016年主要工作安排

　　今年是全面建成小康社会决胜阶段的开局之年，是推进供给侧改革、全面实施“调转促”行动计划的攻坚之年，是铜陵建市六十周年，也是“一江两岸”新铜陵扬帆起航第一年。我们要认真贯彻落实中央、省系列会议和市委九届十六次全会精神，积极适应和引领经济发展新常态，坚持改革开放，坚持稳中求进工作总基调，坚持稳增长、调结构、惠民生、防风险，着力组织实施好“生态建设提升年”、“规划引领突破年”、“精准扶贫和棚改攻坚年”三个年活动，推动经济社会稳定健康发展，努力实现“十三五”发展的良好开局。

　　今年经济社会发展的主要预期目标是：全市生产总值增长8.5%以上；财政收入增长3%；全社会固定资产投资增长11%以上；社会消费品零售总额增长10%；城镇、农村居民人均可支配收入分别增长8.5%和10%；居民消费价格涨幅3%左右；城镇登记失业率控制在4%以内；完成省政府和国家节能减排示范市确定的年度目标任务。在实际执行中，努力好中求快，好中求优，以优为本，能快则快。

　　重点抓好八个方面工作：

　　（一）聚焦供给侧改革，在稳定经济增长上取得新突破。围绕提高供给结构适应性和灵活性，认真落实优化存量、引导增量、主动减量各项措施，去产能，去库存，去杠杆，降成本，补短板，推动经济平稳健康较快发展。

　　积极化解过剩产能。严格环保、能耗、技术标准，淘汰落后产能。按照企业主体、政府推动、市场主导、依法处置的办法，对资不抵债、扭亏无望的“僵尸企业”，采取一企一策，通过兼并重组、债务重组和破产清算，实现市场出清。落实不良资产处置、财政专项奖补等支持政策，做好失业人员再就业和生活保障，尽可能多兼并重组，少破产清算，保持社会稳定。

　　推进企业降本增效。全面落实减税降费政策，进一步清理各种不合理收费特别是垄断性中介服务收费，降低企业税费负担和各项成本。进一步规范优化政务服务和中介服务，降低制度交易成本。深化“暖企行动”，每户规上企业确定一名县处级领导干部对口联系，建立常态化企业帮扶机制，着力稳定产品有市场、有效益但暂时有困难的企业。整合财政扶持发展专项资金，规范发展三支基金，发挥引导和撬动发展作用。

　　着力化解房地产库存。落实有利于促进住房消费的各项政策。以满足新市民住房需求为出发点，建立购租并举的住房保障制度，鼓励进城农业转移人口就地购房或长期租房，进一步释放住房刚性需求和改善性需求。在棚户区改造、房屋征收过程中，加大货币化安置力度。同时，合理管控增量，把握好土地供应节奏，多措并举促进房地产市场稳定发展。

　　推进经济金融良性互动。坚持促发展与防风险并举，引导金融机构落实企业续贷政策，深化政银担合作，扩大“税易贷”等业务规模，全年力争新增贷款60亿元。加大与资本市场对接力度，努力实现主板上市“破冰”，新增“新三板”挂牌企业5家、“新四板”挂牌企业10家左右，力争完成直接融资100亿元。着力防范化解金融风险，加强政府债务管理制度的落实，继续做好政府存量债务置换工作。加强信用体系建设，坚决打击非法集资的行为，健全重大风险处置协调机制，维护良好的金融生态环境。

　　持续扩大有效投资。聚力重大项目建设，强化落实“四督四保”制度，全年安排亿元以上项目超500个，其中新开工100个、竣工80个，完成投资400亿元。加快推进有色“奥炉”项目建设，争取第一批新能源汽车整车下线，促进皖能第二台百万千瓦机组、艾默生家电连接器、全威压延铜箔、新能源电池组等一批项目竣工投产。对应中央预算内投资重点支持领域，制定全市项目谋划、争取工作清单，做细做实项目前期，做好筛选申报，提高“入围率”。

　　振兴县域经济和园区经济。出台支持县区经济发展意见，完善分类考核办法，赋予县区更多自主权，激发县区发展动力和活力，实现增长速度高于全市平均水平，发展位次省内前移。实施“园区倍增计划”，理顺园区管理体制机制，引导园区壮大特色主导产业，落地建设一批新能源、新材料、智能化等重大项目。推动铜基新材料集聚基地建设取得突破性进展，市经开区生产总值增长10%以上。力争狮子山国家级高新区获批。推进枞阳经济开发区转型发展。促进示范园区现代物流业发展取得显性进展。

　　（二）聚焦产业集聚，在推动“调转促”上取得新突破

　　坚持以创新引领发展。抓住我省系统推进全面创新改革试验机遇，加快创新型试点城市建设。强化企业创新主体地位，鼓励企业申报建设技术创新平台，新增省级以上研发机构3家，新认定国家高新技术企业15家以上，争取PCB质检中心成为国家级中心。深化产学研协同创新，重点支持铜基新材料等领域关键技术研发。积极引进领军人才和创新团队，扩大高层次人才股权、分红激励试点，打通科技向现实生产力转化通道。大力推进大众创业、万众创新，持续培育一批众创空间和专业孵化器。争创国家质量强市示范市。

　　促进产业迈向中高端。大力改造传统优势产业。认真实施“千百十亿”工程，启动新一轮技改升级计划，推进“互联网+制造”，建设一批“数字车间”、“智慧工厂”和智能制造示范项目。加快培育战略性新兴产业。整合资金等要素资源，高水平推进铜基新材料、新能源汽车、智能装备、精细化工等产业集聚发展，努力抢占产业发展制高点，积极申报省第二批新兴产业集聚基地。

　　提质发展现代服务业。完善政策扶持体系，大力发展金融、现代物流、检验检测和科技服务等生产性服务业，推动健康、养老等生活性服务业向精细化、高质化转变，引导流通企业线上线下融合发展，培育互联网产业。积极参与皖南国际文化旅游示范区建设，推动环天井湖景区争创国家级旅游休闲度假区，浮山景区争创国家5A级景区，凤凰山景区、大通古镇风景区争创国家4A级景区，发挥高铁旅游联动效应，打造黄山、九华山重要的中转旅游地。培育文化新业态，推动文化产业健康发展。

　　（三）聚焦开放合作，在发展外向型经济上取得新突破

　　加大招商引资力度。坚持引资引智一体化，精准推进产业链招商、产业板块招商和以商引商，高效对接央企、知名民企和外资企业，着力在招大引强上取得突破，不断掀起招商引资热潮。全年省外投资亿元以上项目到位资金420亿元，引进外商直接投资2.3亿美元，每个县区引进超10亿元的产业项目2个以上。做好引资企业的服务，提升投资便利化水平，优化营商环境。

　　积极参与长江经济带建设。开工建设南夹江综合治理工程，配合做好引江济淮项目建设，完成G3高速铜陵长江替代桥前期工作。推动合肥铜陵经济带建设，启动江北长江岸线整治，谋划推进江北港区建设，打造合肥经济圈通江达海新通道。改造升级横港等老港区，加快永丰等港区公用码头和服务基地建设。建成进境水果口岸，建设粮食口岸。尽快运营港口集团，广泛开展与沿江沿海口岸合作，提升江海联运、多式联运水平，成为亿吨大港，促进港口经济跨越式增长。

　　稳定外贸增长。完善促进外贸发展的综合政策，鼓励引导本地外贸企业属地报关，支持骨干外贸企业发展，培育外贸新主体，大力发展服务外包，外贸进出口完成46亿美元。发挥“圈区管理”园区综合效益，争取成为进口废七类集装箱转关转检第一口岸。创造条件积极申报综合保税区。融入“一带一路”战略，加快“走出去”步伐，引导企业开拓境外市场。

　　（四）聚焦“三农”工作，在发展现代生态农业上取得新突破

　　提升农业综合生产能力。落实藏粮于地、藏粮于技战略，实行最严格的耕地保护制度，提高粮食绿色增产水平，支持枞阳县再创全国粮食生产先进县，力争粮食总产稳定在70万吨以上。努力抓好农田水利基本建设，加强长江崩岸应急治理，实施中小河流治理和病险水库加固工程，新增高标准基本农田2万亩。深入实施“菜篮子”工程，创建一批省级以上蔬菜、畜禽养殖示范基地。运用现代技术改造传统渔业，打造农业经济重要增长极。

　　构建现代农业产业体系。培育壮大农业产业化龙头企业，做优油用牡丹、白姜、水产等特色农产品，建设一批产业加工区、休闲观光区，加快发展“一村一品”，提升农业综合效益，增加农民收入。促进一二三产业深度融合发展，加快粮经饲统筹、农林牧渔结合、种养加一体。推动普济圩农场向大型农工商产业集团迈进。加快培育新型经营主体，规范农民专业合作社和家庭农场建设。引导土地承包经营权有序流转，促进适度规模经营。加强农民工职业技能培训，培育新型职业农民。

　　推进镇村建设。以“三治三增三提升”行动为抓手，扶持和培育陈瑶湖、横埠、大通和钟鸣等一批重点镇，打造县区经济社会发展次中心。按市区标准，提升老洲镇管理水平。以枞阳、义安为重点，抓好美丽乡村建设，建成25个省级中心村、18个市级中心村。集中开展乡镇政府所在地中心村环境整治，全面推进农村公路畅通工程，改善乡村人居环境。

　　（五）聚焦重点领域，在全面深化改革上取得新突破

　　深化行政体制改革。坚持“放、管、服”结合，建立政府权责清单动态发布机制，做好上级下放审批事项的承接。制定政务服务上网目录，建成运行市社会服务信息化平台，让信息多跑路、公众少跑腿。扩大商事制度改革成果，开展注册登记电子化试点。加强招投标管理改革，整合市、县公共资源交易平台。

　　推进国企国资改革。积极稳妥推进铜化集团混合所有制改革。推动市建投市场化转型，增强经营城市能力。提升铜陵发展投资风险防控和国有资本投资运营能力。加强国有资产归集和监管，大力盘活闲置资产，进行市场化运营，实现保值增值。

　　大力推进财税金融体制改革。健全全面规范、公开透明的预算制度。推进市县事权和支出责任界定，完善县区政府财力保障机制。落实税制改革任务，深化税收征管体制改革。加强政府与社会资本合作，推动城市排水一体化PPP试点运营，再推出停车场、地下管廊、城市道路等一批合作项目，激发社会资本投资热情。

　　加快推进社会事业改革。巩固教育综合改革成果，促进跨区联合办学，扩大优质教育资源覆盖面。深化医药卫生体制改革，推动医疗联合体扩面提升，争创综合医改示范市。探索大病医疗保险、城乡居民医疗保险商业化运营。推进社会组织规范化发展，加快政社脱钩。

　　深入推进城乡一体化综合配套改革。完善城乡一元化户籍制度，加快农业转移人口市民化。深化农村产权制度改革，基本完成农村土地承包经营权确权登记颁证试点任务，扩大农村产权抵押融资成果，让资源变为资本。深化供销社综合改革。推进国有农场和林场集团化改革。实现不动产登记统一发证。

　　（六）聚焦融合联动，在区域协调发展上取得新突破

　　强化规划引领。坚持把“一尊重、五统筹”要求贯穿城市发展全过程，构建以长江为龙头、南北为两翼的城市发展格局。编制城乡发展战略规划，修编新一轮城市总体规划、土地利用总体规划、环境保护规划。组织修编市域综合交通、港口岸线利用、水利防洪、产业布局、生态功能区保护、市政和公共服务设施专项规划。审批枞阳县城市总体规划。推动“多规合一”正式运行。加强城市设计，进一步塑造城市特色。坚持“规划即法、执行必严”，严格规划监督，严查各类违反规划行为，确保规划的权威性和严肃性。

　　推进综合交通体系建设。开工建设国道347工程，推进麒麟至白湖二级公路建设，力争完成国道237、330和省道425一级路改建前期工作。推动济祁高速、北沿江高速尽快开工建设。实施铜芜路一级公路改建，建成坝白路、站前路二期，开工建设胥坝跨江大桥，启动顺安河三级航道升级工程。积极配合庐铜铁路、芜铜铁路电气化改造项目建设，谋划推进过江通道、铜九黄轨道交通、铜宣城际铁路等项目。

　　完善新城区功能。推动优质教育、医疗资源向新区布局，建成西湖公立示范幼儿园，实行市区一流的中小学与新区学校联合办学，实现市一中西湖校区秋季开学。加快建设市儿童医院。建成西湖现代农业生态休闲示范园，年中向市民开放。加快东部城区、高铁新区配套设施建设。提升南部城区生态环境和城区功能。支持枞阳新区建设。

　　提升老城区品质。开工建设大铜官山公园。结合地下综合管廊建设，改造长江东路、长江中路。建成一批城市微循环道路。大力推进停车场和电动汽车充电桩建设，实施公交智能化工程，继续投放一批清洁能源公交车。建设市档案馆、史志馆和城建档案馆，建成市烈士纪念馆。继续改造一批老旧小区。

　　加强城市管理。精心精细管理城市，推动扁平化、网格化管理向全市域覆盖，向基层延伸，完善区直接管理社区体制。深入推进国家智慧城市、“宽带中国”示范市建设，加快光纤入户进程。推动城市综合管理精细化、智能化。打造“数字城管”。巩固全国文明城市建设成果，加强烟花爆竹燃放管理。

　　（七）聚焦绿色发展，在生态文明建设上取得新突破

　　加强环境综合治理。着力治理大气污染，加快实施国电、皖能燃煤发电机组超低排放改造，重拳整治南部城区、港区和工矿区扬尘污染，巩固治超治泼和秸秆禁烧成果，落实黄标车区域禁行制度，强化餐饮油烟和挥发性有机废气治理，力争PM10平均浓度下降4%。着力治理水体污染，向城市黑臭水体宣战。把长江岸线生态保护和修复摆在压倒性位置，开展中心城区六大入江排污口整治专项行动，全面提高城区、园区和企业污水处理能力，以企业的达标排放保证流域水质达标，保护好长江“母亲河”。着力治理土壤污染，严控重金属源头排放，开展土壤污染综合治理与修复试点。强化问题导向，狠抓环保督查问题整改。落实各级环保责任，严格问责问效。深化环境监察网格化、精细化管理，强化联合执法，严厉打击环境违法行为。

　　提升生态建设水平。落实主体功能区战略，划定城市发展边界线，严守生态保护红线。推进海绵城市建设，优化城市绿地、广场、排水、截污等生态调节功能，构建完善城市水生态系统。深入创建国家森林城市，持续实施森林增长和绿化提升工程，人工造林2.1万亩，改造提升绿化250万平方米，新增森林长廊示范段15公里、城市绿道40公里，新建街头绿地（游园）18个，恢复矿山植被2000亩，基本达到国家森林城市标准。巩固“三线三边三小”整治成果，延长滨江生态生活岸线。

　　推进资源节约高效利用。开展全民节能行动，全面完成国家节能减排示范市建设任务，争取在全国继续保持优秀等次。深入推进重点产业循环化改造，做好循环经济示范市创建工作。推进国家工业绿色转型发展试点市建设。巩固全国节水型示范市建设成果。坚持节约用地，推进低效用地再开发和工矿废弃地复垦。

　　（八）聚焦扶贫与棚改，在改善民生上取得新突破。牢固树立过"紧日子"的思想，把有限的财力更多用在保障和改善民生上，精心实施38项民生工程，保基本、兜底线、促公平，继续走在全省前列。

　　强力推进精准扶贫攻坚。按照"三年攻坚脱贫、两年巩固提升"的目标，制定落实扶贫开发"四项清单"，强力推进脱贫攻坚"十大工程"，全年减少贫困人口1.9万人。推进定点扶贫工作，建立领导联系和单位帮扶贫困制度，实行"单位包村、干部包户"和驻村工作队定点帮扶。推进市辖区与贫困地区产业层面合作。鼓励社会力量参与，激励企业、社会组织、个人自愿参与扶贫，汇聚脱贫攻坚的强大合力。

　　大力实施棚改攻坚。坚持滚动推进、持续发力，实施扫把沟地区、东村、狮子山老城区、市一中周边、义安城北等片区棚户区改造，完成搬迁改造6000户以上，实现棚户区改造三年任务大头落地。持续加大回迁安置力度，新安置居民1万户以上，基本解决历史遗留的安置问题。

　　提升就业和社会保障工作水平。坚持以创业带动就业，争创国家创业型城市，新增就业2.5万人。落实全民参保登记计划，扩大社会保险覆盖面。全面实施机关事业单位养老保险制度。出台市县统一的城乡居民医保政策。加大社会临时救助和医疗救助力度，提升"救急难"试点市成效。完善养老服务体系。加强社区养老服务设施建设与运营。加大养老机构建设力度，探索公办养老机构社会化运营，支持民办养老机构加快发展。启动建设医养结合型养老中心。

　　统筹发展各项社会事业。努力办好人民满意教育，下决心解决群众上好学校难问题。推进教育现代化。加快发展学前教育，推动义务教育阶段学校标准化建设，加快普及高中阶段教育，深化职业教育产教融合、工学结合和校企合作，推动铜陵职业技术学院建设地方技能型高水平大学，支持铜陵学院建设具有鲜明特色的应用型高水平大学。加强继续教育和特殊教育，支持和规范民办教育。加快建设国家公共文化服务体系示范区。推进文化惠民，加大对地方文化建设的投入力度，组织实施七大类文化项目。做好铜文化、桐城派文化等优秀传统文化的研究、保护和传承，举办第十三届中国（铜陵）青铜文化博览会。办好2016年中国图书馆年会。健全医疗卫生服务体系，提高医疗单位服务能力和服务水平，实施名院名医名科战略，支持市"三甲"医院向枞阳辐射，提升皖中南医疗服务中心地位。落实好"全面两孩"政策。广泛开展全民健身运动。推进中央广播电视无线数字化工程，实现节目信号全域高质量覆盖。加快发展妇女儿童事业。大力发展社会福利和慈善公益事业。做好残疾人基本公共服务和权益保障。

　　加强和创新社会治理。大力培育和践行社会主义核心价值观，广泛开展志愿服务。提升社区治理和服务水平，完善社会矛盾排查预警和调处化解机制。推进网上信访，集中化解信访积案。开展安全生产"铸安"行动，推进事故隐患排查治理全覆盖，坚决遏制较大以上安全事故发生。严格食品药品安全监管，争创省食品药品安全城市。加强引导、妥善应对网络舆情。健全应急管理机制，提高突发事件应急处置能力。加强气象、地质、地震、防灾减灾工作。启动"七五"普法，推进法治铜陵建设。建设"长安"城市，完善社会治安防控体系，依法严厉打击各类违法犯罪活动，维护社会大局和谐稳定。

　　四、加强政府自身建设

　　把握新常态，建设新铜陵，对各级政府特别是领导干部提出了更高要求。我们要强化"忠诚、干净、担当"意识，认真践行"三严三实"，勇担历史使命，顺应群众期待，全面提高施政水平，努力建设人民满意政府。

　　（一）建设法治政府。要认真贯彻《法治政府建设实施纲要》，依法全面履行政府职能。要加强政府立法工作，制定地方规章规划，严格规范性文件管理。要健全依法决策机制，把公众参与、专家论证、风险评估、合法性审查和集体讨论决定作为法定程序，充分发挥政府法律顾问的作用，提高行政决策的科学化、民主化、法治化水平。要深化行政执法体制改革，推进执法重心下移，推行综合执法，严格执法程序，创新执法方式，全面落实行政执法责任制。做好行政复议、行政应诉工作。要自觉接受人大法律监督、工作监督和政协民主监督，认真办理人大代表建议和政协提案。广泛听取各民主党派、工商联、无党派人士和人民团体意见，主动接受社会监督、舆论监督。把政务公开贯穿政务运行全过程，推进财政预算、公共资源配置、重大项目和社会公益事业建设等领域的信息公开，落实政府发言人制度，加强公开平台建设，以公开促落实、以公开促规范、以公开促服务。

　　（二）建设学习型政府。学习既是一项工作，更是一种责任。面对新形势、新市域、新任务，各级政府特别是领导干部更要加强学习，坚持以上率下，切实解决能力不足、"本领恐慌"问题。要学习新理论。深刻领会习近平总书记治国理政思想，自觉践行五大发展理念，增强看齐意识，争当"四个自觉"的模范。要学习新政策。准确把握上级政策导向、政策逻辑，摆脱传统路径依赖，让新的发展理念在铜陵落地生根。要学习新市情。破除陈旧观念，颠覆惯性思维，无论是作决策、定政策，还是出主意、提建议，都要树立大市域观念，增强统筹意识。要学习新知识，做到开阔视野，与时俱进。各级领导干部要发扬刻苦钻研精神，干中学，学中干，努力成为各领域的行家里手。

　　（三）建设担当政府。政府必须有为，为官必须担当。要坚持守土有责、守土负责、守土尽责，保持奋发向上的工作激情，直面矛盾，正视问题，善谋善断，敢做敢为。要大力弘扬真抓实干的工作作风，发扬"钉钉子"精神，善做善成，"深、实、细、严、快"地谋划和推进工作，无论是目标任务，还是政策措施，定了就干、干就快干，干就干好。要强力治庸治懒治怠，强化干部问绩问效，对不作为、乱作为、慢作为的，要划红线，严问责。要强化目标考核管理，优化评价指标体系，完善落实的责任机制、奖优罚劣的激励机制和常态的督查机制，营造竞相有为、主动作为的干事创业环境，形成领着干、比着干、推着干的浓厚氛围。要坚持以人民为中心，进一步健全为基层、为群众服务的各项制度，加强政务服务中心、网上办事大厅、市长热线、行风热线平台建设，一诺千金，全面提升服务效能。

　　（四）建设清廉政府。要严格落实中央八项规定，坚持勤俭节约，严控"三公"经费支出，弛而不息转作风。要不懈改进和加强政府管理，强化对薄弱领域和关键环节权力运行的风险防控，对公共资金、公共资源、国有资产实行常态化监管，扎紧制度笼子。要深入推进政府系统党风廉政建设，落实"两个责任"，强化"一岗双责"，加强行政监察，推进审计监督"四个全覆盖"，坚决查处发生在群众身边的"四风"和腐败问题，保持反腐败的高压态势。各级领导干部要带头遵守廉洁自律准则，严格廉洁从政、廉洁用权、廉洁修身、廉洁齐家，树立清正廉洁的良好形象。

　　各位代表！新的目标鼓舞人心，新的任务光荣艰巨，新的征程催人奋进。让我们紧密团结在以习近平同志为总书记的党中央周围，在省委、省政府和中共铜陵市委的坚强领导下，开拓进取，扎实苦干，为打造"四个富有"城市，建设幸福美丽新铜陵而努力奋斗！
